# The Number of Offspring Weaned from Ewe Lambs Is Affected Differently by Liveweight and Age at Breeding

**DOI:** 10.3390/ani11092733

**Published:** 2021-09-18

**Authors:** Andrew N. Thompson, Elise Bowen, John Keiller, Don Pegler, Gavin Kearney, Cesar A. Rosales-Nieto

**Affiliations:** 1 Centre for Animal Production and Health, Murdoch University, Murdoch, WA 6150, Australia; sheepdatamanagement@gmail.com; 2 Cashmore Park, 114 Wilmots Road, Cashmore, VIC 3305, Australia; cashmoreram@gmail.com; 3 Oaklea Genetics, 88 Meyers Road, Nene Valley, SA 5291, Australia; pegler4@bigpond.com; 4 36 Payne Road, Hamilton, VIC 3300, Australia; gke29755@bigpond.net.au; 5 Facultad de Agronomía y Veterinaria, Universidad Autónoma de San Luis Potosí, San Luis Potosí 78321, Mexico; nieto_cesar@hotmail.com

**Keywords:** ewe lambs, breeding liveweight, breeding age, reproductive rate, weaning rate

## Abstract

**Simple Summary:**

Ewe lambs can reach puberty and conceive at 7 to 10 months of age and those that are heavier at breeding are consistently more fertile. The aim of this research was to quantify the separate effects of age and liveweight at the start of breeding on the components of weaning rate. The analysis of data from more than 11,500 maternal composite ewe lambs indicated that ewe lambs that were heavier at the start of the breeding period weaned more offspring than lighter ewes, but if ewe lambs reached 45 kg their weaning rate was within 5% of their maximum for a given age. By contrast, the effects of age at breeding on weaning rate was linear and increased by 0.4% per day. Within the range from 35 to 45 kg liveweight and 6 to 9 months of age, a 1-kg increase in the liveweight at the start of breeding had the equivalent effect on weaning rate as an extra 7 days of age at the start of breeding. This understanding of the trade-off between age and liveweight at breeding will assist farmers to optimize the management and reproductive performance of ewe lambs.

**Abstract:**

In this paper, we tested the hypothesis that ewe lambs that are heavier and older at breeding will wean more offspring, due to increased reproductive rate and offspring survival and lower maternal mortality. To test this hypothesis, we analyzed data from more than 11,500 maternal composite ewe lambs collected over eight years. The ewe lambs had full pedigree records including birth type, age and liveweight at breeding plus records of the birthweight and survival of their offspring and the dam. The average liveweight and age at breeding was 40.2 kg and 228 days. The reproductive rate and weaning rate responses to liveweight at breeding were curvilinear (*p* < 0.001), and if ewe lambs achieved 45 kg by the start of breeding, their reproductive rate and weaning rate were within 5% of their maximum. By contrast, the effects of age at breeding on weaning rate was linear and increased by 0.4% per day, despite a quadratic (*p* < 0.01) effect of age at breeding on reproductive rate which increased only marginally when ewe lambs were older than 8 months at breeding. Increasing liveweight (*p* < 0.05) or age (*p* < 0.001) at breeding increased survival of their offspring, however an extra 10 kg of liveweight or 30 days of age at breeding increased offspring survival by less than 5%. Both liveweight (*p* < 0.001) and age (*p* < 0.01) at breeding also influenced survival of the ewe lamb dam but survival rates exceeded 95% across the range in liveweights from 30 to 55 kg and ages from 6 to 9 months. This understanding of the trade-off between age and liveweight at breeding will assist farmers to optimize the management of their ewe lambs, given the earlier they can be bred successfully the easier they can be integrated with the breeding of the adult ewe flock the following year.

## 1. Introduction

Breeding ewe lambs at 7 to 10 months of age can increase lifetime reproductive performance [1,2] and farm profitability [3,4,5]. The number of offspring weaned from ewe lambs is highly variable and generally poor in comparison to older ewes [6,7,8,9]. Ewe lambs that are heavier at breeding due to improved nutrition pre- and post-weaning consistently achieve higher fertility and reproductive rates, although the responses vary significantly between individual flocks and breeds [10,11,12,13,14,15]. Improved nutrition during the breeding period is also positively correlated to reproductive rate, and the effects of growth rate during the breeding period were evident irrespective of the liveweight at the start of breeding indicating that their effects are additive [13,14]. By contrast, there is limited evidence regarding the effects of liveweight at breeding, when 7 to 10 months of age, on other components of weaning rate including the survival of their offspring. Griffiths et al. [16] reported that liveweight at the start of breeding had no effect on the likelihood of a ewe lamb failing to rear a lamb to weaning, however survival of the offspring was not quantified. Offspring from ewe lambs with higher breeding values for post-weaning weight were heavier at birth [17], and birthweight is critical to the survival of offspring from ewe lambs given they tend to be significantly lighter at birth than the offspring from adult ewes [2,6,7]. There are also no data regarding the effects of liveweight at breeding on the mortality of the ewe lamb. However, it could be expected that the survival of ewe lambs and their offspring will be greater when ewe lambs are heavier at breeding.

Generally, increasing age at breeding is associated with higher fertility and reproductive rate [18,19]. Gaskin et al. [19] reported that varying age at breeding influenced the probability of ewe lambs becoming pregnant but the effect was variable and age had no effect on the probability of multiple births. Furthermore, the studies of Dyrmundsson [6], Laster et al. [18] and Gaskin et al. [19] used breeds not common to the sheep production systems in Australia, including Targhee, Columbia, Rambouille, Polpay and Finn crosses, the number of ewe lambs was limited to between 7 and 630 per breed and most of the ewe lambs were between 6 and 7.5 months old during breeding. In addition, there appears to be no evidence regarding the effects of age at breeding on the survival of the ewe lamb and her offspring, and the effects of age and liveweight at breeding are often confounded and there have been few attempts to separate their effects. If ewe lambs can be bred successfully at a younger age they can be more easily integrated with the breeding of the adult ewe flock the following year. Understanding the trade-offs between age and liveweight at breeding on weaning rate and its components will therefore assist sheep producers to optimize decisions related to the time of breeding ewe lambs and their nutritional management before breeding through to lambing. Therefore our study tested the hypothesis that ewe lambs that are heavier and older at breeding will wean more offspring, due to increased reproductive rate and offspring survival and lower maternal mortality. To test these hypotheses, we analyzed data from more than 11,500 maternal composite ewe lambs sourced from commercial ram breeders collected from 2009 to 2017.

## 2. Materials and Methods

### 2.1. Data Source

The current study was undertaken on two commercial farms in Australia, Cashmore Park located in southwestern Victoria (38.31° S, 141.48° E) and Oaklea Genetics located in southeastern South Australia (37.56° S, 140.29° E). Animals used in the analysis were maternal composite ewe lambs and the data consisted of 11,599 records from ewe lambs bred in 2010 to 2017 (Table 1). The mix of breeds within the maternal composite was 53% Coopworth, 20% White Suffolk, 10% Poll Dorset, 7% Border Leicester, 5% Texel, 2% East Friesian, 2% Romney and 1% Finn. All data were collected as per normal farm practice over the period from 2009 to 2017. The data were sourced directly from the ram breeders and from LambPlan, the genetic evaluation system for terminal and dual-purpose sheep producers in Australia [20].

### 2.2. Animal Management and Measurements

The following procedures were performed each year with only minor variations between years and farms. The dams of the ewe lambs used in this study were pregnancy scanned by transabdominal ultrasonography about 50 days after the end of breeding and separated into mobs of dry, single, twin and triplet bearing ewes. For each birth type, there was a single mob of adult ewes (1¾ to 6¾ years old) and a single mob of ewe lambs (¾ years old). The adult ewes were differentially fed during mid and late pregnancy to achieve body condition score targets at lambing of 2.7 to 2.8 for singles, 3.1 to 3.3 for twins and 3.4 to 3.5 for triplets. The condition score targets were less well defined for the ewe lambs, but the multiple bearing ewe lambs were preferentially fed compared to the single bearing ewe lambs. The ewe lambs commenced lambing about one-month after the adult ewes.

Prior to lambing, the single, twin and triplet bearing adult ewes were split into mobs of 150 to 250, 70 to 80 and 30 to 40, respectively. The single and multiple bearing ewe lambs were split into mobs of 120 to 150 and 70 to 80 for lambing. All offspring were tagged within 24 h of birth and their birth date, dam, sex, birth type, birth weight and survival at birth was recorded. From marking to weaning, all single bearing adult ewes and their offspring were combined into one mob, twin bearing adult ewes and their offspring were combined into one to three mobs and triplet bearing adult ewes and their offspring were combined into one mob. Single and multiple bearing ewe lambs and their offspring were managed as single mobs. Any dry ewes (failed to rear any offspring) were removed at marking.

All offspring were weighed at weaning and offspring that were tagged at birth but not present at weaning (mean age 84 days) were classified as dead. No ewe lambs were culled other than when their welfare was compromised. From weaning to breeding, all ewe offspring from adult ewes were managed together and the same applied for ewe offspring from the ewe lambs.

In most years ewe lambs were weighed at the start of breeding but in some years they were weighed 2 to 4 weeks pre-breeding. When this occurred their liveweight at the start of breeding was calculated assuming linear growth between the pre-breeding liveweight and another weight collected at the end of breeding or soon after. Ewe lambs were naturally bred with a single ram at a ratio of 100 to 120:1 for 42 days and the target growth rate during breeding was 100 to 200 g/day depending on seasonal conditions. The ewe lambs were not teased with vasectomized rams prior to the introduction of the entire rams. After breeding, all ewe lambs were managed as a single mob until pregnancy scanning. Management and measurements from pregnancy scanning onwards were as described earlier, with the addition that the identification of any ewe lambs that died during late pregnancy, lambing or lactation was recorded.

### 2.3. Statistical Analysis

Data were analyzed by the following methods using GENSTAT (Edition 19 [21]). The Method of Restricted Maximum Likelihood was used to fit ewe liveweight and age at breeding with birth type of the ewe lamb as fixed effects while year was fitted as a random effect. Reproductive rate and weaning rate were analyzed using a Generalized Linear Mixed Model with a multinomial distribution and logit link function as a function of different variables, including liveweight and age at breeding and birth type of ewe lamb as fixed effects and year as a random effect. For liveweight and age at breeding, quadratic effects were also examined.

Estimates of ewe survival were assessed by fitting Generalized Linear Mixed Models. The approach used a logit transformation and binomial distribution. Using additive models, logits were predicted as a function of birth type of ewe lamb, liveweight and age at breeding and pregnancy status fitted as fixed effects while year was fitted as a random effect. Both ewe liveweight and age at breeding were tested for quadratic effects.

Estimates of offspring survival were assessed by fitting Generalized Linear Mixed Models. The approach used a logit transformation and binomial distribution. Using additive models, logits were predicted as a function of lamb birth weight (quadratic effect), ewe liveweight and age at breeding, the ewe lambs’ own birth type, offspring birth type and sex fitted as fixed effects while year and sire of offspring (nested within year) were fitted as random effects.

The Method of Restricted Maximum Likelihood was used to fit offspring birth weight data with liveweight and age of the ewe at breeding, birth type of ewe lamb and birth type and sex of offspring fitted as fixed effects where appropriate while year and sire of offspring (nested within year) were fitted as random effects.

All possible models in the analysis described above were examined with statistical significance of terms and interactions thereof accepted at *p* < 0.05.

## 3. Results

### 3.1. Liveweight and Age at Breeding of Ewe Lambs

The average liveweight of the whole flock at breeding was 40.2 kg and this varied between years from 38.0 to 43.4 kg (Table 2). The liveweight at breeding was also influenced by the birth type of the ewe lambs (*p* < 0.001), and on average those born as singles were heavier than those born as twins or triplets (42.3 vs. 39.7 and 38.6 kg). The average age of the whole flock at breeding was 228 days and this varied between years from 221 to 235 days and it was also influenced by birth type (*p* < 0.001; Table 2). On average, ewe lambs born as singles were younger at breeding than those born as twins or triplets (222 vs. 229 vs. 236 days), because the multiple born ewe lambs were born one to two weeks earlier than the single-born ewe lambs. The average age of conception, estimated from the birth date of the offspring from ewe lambs and assuming a 150-day gestation, was 25 days after the start of breeding. Across all 11,599 ewe lambs in the study, over 60% were in the range 35 to 45 kg liveweight and 6 to 9 months of age at the start of breeding.

### 3.2. Reproductive Performance and Survival of Ewe Lambs

The average fertility rate, reproductive rate and weaning rate of the whole flock was 76.4, 117.4 and 76.9%, respectively. This varied between years from 58 to 93% for fertility rate, 95 to 145% for reproductive rate and 58 to 102% for weaning rate (Table 3). Reproductive performance appeared to increase over the eight years from 2010 to 2017. On average, there was no effect (*p* > 0.05) of the ewe lambs’ own birth type on fertility, however reproductive rate and weaning rate were lower for ewe lambs born as singles (110.4 and 69.1%) compared to those born as twins (115.8 and 76.1%) or triplets (121.1 and 80.1%).

The overall survival of the whole flock between breeding and the weaning of their offspring was 96.7% and this varied between years from 93.7 to 98.7% (data not shown). The rate of survival of ewe lambs was not influenced by the ewe lambs’ own birth type (*p* > 0.05) nor whether the ewe lamb was scanned to be carrying single or multiple fetuses (*p* > 0.05).

### 3.3. Offspring Birthweight and Survival

The proportion of single-, twin- and triplet offspring born to ewe lambs was 28, 67 and 5%, respectively. The average birthweight of the offspring from ewe lambs was 4.3 kg, and this varied from 3.8 to 4.6 kg between the years (Table 4). The single-born offspring were heavier at birth than the twins or triplets (4.9 vs. 4.2 vs. 3.6 kg; *p* < 0.001), and the male offspring were consistently heavier at birth than the females (4.5 vs. 4.2 kg; *p* < 0.001). The birth weights were slightly greater for offspring from twin- compared to single-born ewe lambs (4.43 vs. 4.37 kg, *p* < 0.05).

The average survival of offspring from ewe lambs was 68%, and this varied from 61 to 72% between years. Survival was influenced by the offspring’s birth type (*p* < 0.001) and sex (*p* < 0.001) and the maternal birth type (*p* < 0.01). There was no significant difference in the survival of single- and twin-born offspring, but their survival was much greater than the survival of triplet-born offspring (Table 4). Likewise, the survival of female offspring was significantly higher than the survival of male offspring (76 vs. 57%; *p* < 0.001). Lastly, survival was higher for offspring from ewe lambs born as triplets or twins compared to those from single-born ewe lambs (69% vs. 68% vs. 65%, respectively).

### 3.4. Liveweight and Age at Breeding Effects on Weaning Rate

**Figure 1:**
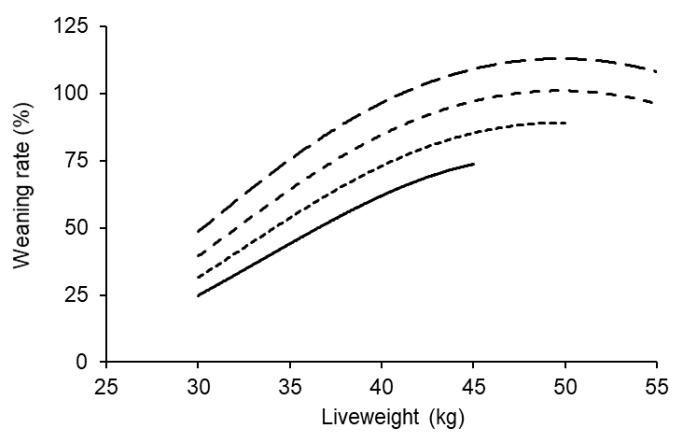
The effect of liveweight at the start of breeding on weaning rate for ewe lambs that were 6 (bottom), 7, 8 or 9 (top) months of age. The data represent over 11,500 maternal composite ewe lambs and the average across the ewe lambs’ own birth type. The average 95% confidence interval across all scenarios was ±6.6%.

Liveweight at breeding was related to weaning rate and the response was curvilinear (*p* < 0.001; Figure 1). The number of offspring weaned per ewe increased by about 3% for each additional kilogram of liveweight at breeding between 35 and 45 kg, and if ewe lambs reached 45 kg at the start of breeding, their weaning rate was within 5% of their maximum for a given age at breeding. The effects of age at breeding on weaning rate was linear and increased by 0.4% per day over the range from 6 to 9 months of age (*p* < 0.001). There was no significant interaction between liveweight and age at breeding, so their effects on weaning rate were additive.

**Figure 2:**
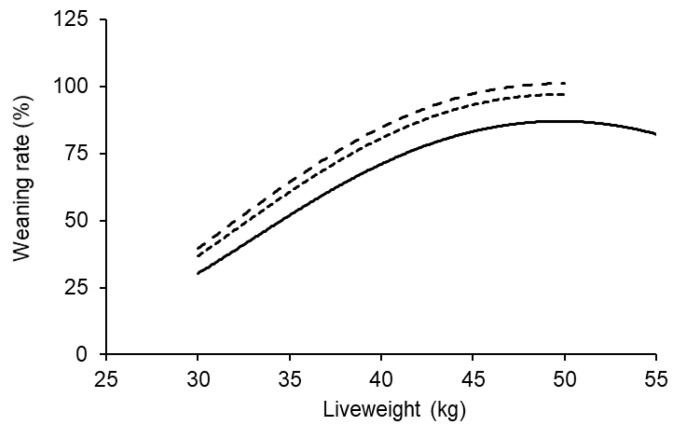
The effect of liveweight at the start of breeding on weaning rate for ewe lambs that were born a single (bottom), twin or triple (top). The data represent over 11,500 maternal composite ewe lambs and weaning rates are predicted at 7.5 months of age. The average 95% confidence interval across all scenarios was ±6.6%.

There was also a significant effect of the ewe lambs’ own birth type on the weaning rate response to liveweight (*p* < 0.001; Figure 2). At the same liveweight and age at breeding, weaning rate was significantly higher for ewe lambs that were born as twins or triplets compared to those born as singles.

### 3.5. Liveweight and Age at Breeding Effects on Reproductive Rate

**Figure 3:**
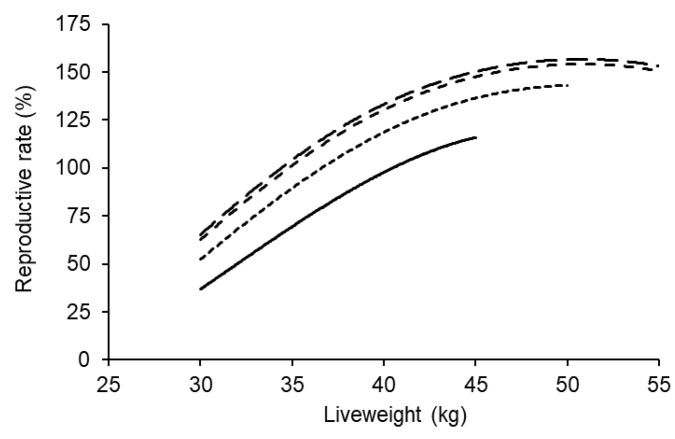
The effect of liveweight at the start of breeding on reproductive rate for ewe lambs that were 6 (bottom), 7, 8 or 9 (top) months of age. The data represent over 11,500 maternal composite ewe lambs and the average across the ewe lambs own birth type. The average 95% confidence interval across all scenarios was ±10.0%.

Liveweight at breeding was related to reproductive rate and the response was curvilinear (*p* < 0.001; Figure 3). Reproductive rate increased by about 4.6% for each additional kilogram of liveweight at breeding between 35 and 45 kg, and if ewe lambs reached 45 kg at the start of breeding, their reproductive rate was within 5% of their maximum for a given age at breeding. There was also a curvilinear effect of age at breeding on reproductive rate (*p* < 0.01). Reproductive rate only increased by a further 2–3% when the ewe lambs were older than 8 months of age at the start of breeding, and there was no difference in reproductive rate between ewe lambs that were 8.5 versus 9 months of age at the start of breeding (Figure 3). There was no significant interaction between liveweight and age at breeding, so their effects on reproductive rate were additive.

**Figure 4:**
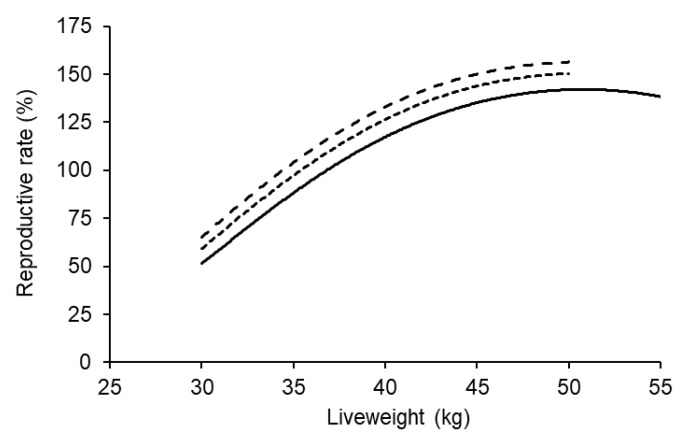
The effect of liveweight at the start of breeding on reproductive rate for ewe lambs that were born a single (bottom), twin or triple (top). The data represent over 11,500 maternal composite ewe lambs and reproductive rates are predicted at 7.5 months of age. The average 95% confidence interval across all scenarios was ±10.0%.

There was also a significant effect of the ewe lambs’ own birth type on the reproductive rate response to liveweight (*p* < 0.001; Figure 4). At the same liveweight and age at breeding, reproductive rate was significantly higher for ewe lambs born as twins or triplets compared to those born as singles.

### 3.6. Liveweight and Age at Breeding Effects on Offspring Birthweight

Ewe lambs that were heavier or older at breeding produced offspring that were heavier at birth (Table 5). Both liveweight and age were significant (*p* < 0.001) when fitted together and each explained additional variance in offspring birthweight, despite liveweight and age being positively related to each other (r^2^ = 0.35; *p* < 0.001). An extra 10 kg of liveweight at breeding increased offspring birthweight by 0.19 kg and an extra 30 days of age at breeding increased offspring birthweight by 0.14 kg. There was also a significant effect of maternal birth type (*p* < 0.05), and birth weights were slightly higher for offspring from ewe lambs that were born a twin or triple after adjustment for differences in their liveweight and age at breeding.

The multiple-born offspring were lighter (*p* < 0.001) than the single-born offspring, and the females were lighter (*p* < 0.001) than the males, but the interactions between liveweight or age at breeding and both birth type and sex were not significant. In other words, the effects of the liveweight and age of the ewe lambs at breeding on offspring birthweight were similar regardless of the birth type or sex of their offspring.

### 3.7. Liveweight and Age at Breeding Effects on Offspring Survival

Birthweight of offspring was strongly correlated with their survival to weaning (*p* < 0.001; Table 6). Survival increased up to a birthweight of 5 kg and only declined marginally for birth weights up to 6.5 kg. Birth type and sex of offspring did not alter the shape of the birthweight versus survival curve (*p* > 0.05), but they did influence the absolute survival at a given birthweight. At the same birthweight, the single-born offspring were about 4% more likely to survive than the twin-born offspring which in turn were about 7% more likely to survive than the triplet-born offspring (*p* < 0.001). The male offspring also had a significantly lower survival than the female offspring at the same birthweight (*p* < 0.001).

Increasing liveweight at breeding increased the survival of the single-, twin- and triplet-born offspring (*p* < 0.05). However, the survival of the single-, twin- and triple-born offspring increased by only 0.4% per kg of extra liveweight at breeding over the range between 35 and 45 kg and there were no further gains in survival of offspring if the ewe lambs achieved 45 kg or more at the start of breeding. Furthermore, when offspring birth weight was added to the statistical model, the effects of liveweight at breeding on offspring survival were no longer significant.

Increasing age at breeding increased the offspring survival for all birth types over the range of 6 to 9 months. The response was linear (*p* < 0.001) and equivalent to 0.16% per day of extra age at breeding. When birth weight of offspring was added to the model, the effects of age at breeding on offspring survival remained just significant (*p* = 0.05; Table 6). The effect of age at breeding on offspring survival was not significant (*p* = 0.14) when maternal birth type was included in the model. Survival was higher for offspring from ewe lambs that were born as multiples, even after adjustment for birthweight, presumably in part because they were older at breeding.

### 3.8. Liveweight and Age at Breeding Effects on Survival of Ewe Lambs

**Figure 5:**
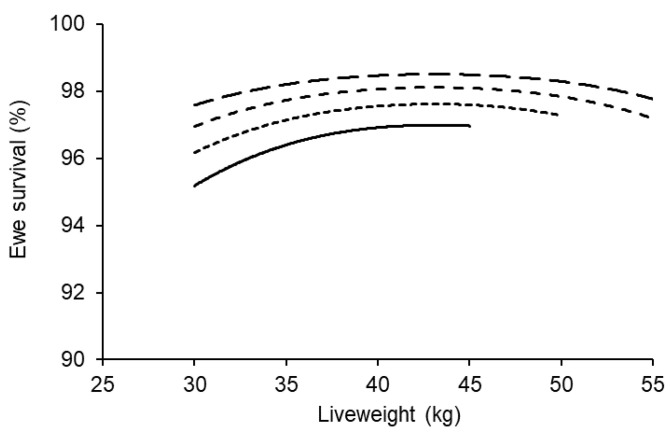
The effect of liveweight at the start of breeding on survival of ewe lambs that were 6 (bottom), 7, 8 or 9 (top) months of age. The data represent over 8800 maternal composite ewe lambs that were pregnant and the average across the ewe lambs’ own birth type and her pregnancy status. The average 95% confidence interval across all scenarios was ±0.7%.

The survival of ewe lambs during late pregnancy, lambing and lactation was influenced by both their liveweight (*p* < 0.001) and age (*p* < 0.01) at breeding (Figure 5). The effect of liveweight was quadratic whereas the effect of age was linear. There was no significant effect of the ewe lambs’ own birth type or her pregnancy status on survival rate.

## 4. Discussion

Maternal composite ewe lambs that were heavier and older at the start of the breeding period weaned more offspring than lighter and younger ewes, which supports our hypothesis. The number of offspring weaned per ewe increased by about 3% for each additional kilogram at breeding between 35 and 45 kg, but if the ewe lambs reached 45 kg at the start of breeding their weaning rate was within 5% of their maximum for a given age at breeding. This response between 35 and 45 kg is double that reported from survey data from New Zealand reported by Kenyon et al. [22], and to our knowledge this is the first study to report the curvilinear effect of liveweight at breeding on the number of offspring weaned from ewe lambs. By contrast to the effects of liveweight at the start of breeding, the effects of age at breeding on weaning rate was linear and increased by 0.4% per day over the range from 6 to 9 months of age. Most of the ewe lambs in the study were in the range 35 to 45 kg and 6 to 9 months. Within those ranges, a 1-kg increase in liveweight at the start of breeding had the equivalent effect on weaning rate as an extra 7 days of age at the start of breeding. The earlier ewe lambs can be bred successfully, the easier they can be integrated with the breeding of the adult ewe flock the following year. This trade-off means that reducing the breeding age of prospective mothers by one month would require them to grow about 30% faster from birth to breeding to avoid compromising the weaning rate of their progeny.

Heavier ewe lambs at the start of breeding weaned more offspring than lighter ewes due largely to their higher reproductive rate rather than the better survival of their offspring. The effect of liveweight at the start of breeding on reproductive rate was curvilinear, so that if ewe lambs achieved 45 kg at the start of breeding their reproductive rate was within 5% of the maximum achieved at heavier liveweights. Curvilinearity of this response to liveweight at breeding has also been reported for other flocks of non-Merino ewe lambs [15,23], although the critical weight beyond which the gains in reproductive rate become marginal varies between 45 and 50 kg. By contrast, in Merino ewe lambs responses are mostly linear at least up to 50 to 55 kg [10,11,12,14,15]. In our study, the reproductive rate increased by about 4.6% per kg between 35 and 45 kg, which is similar or slightly greater than other studies using Merino ewe lambs [10,11,14,15] and more than double that reported for maternal composite, Romney and Coopworth ewe lambs in New Zealand [23]. This variation in the response is presumably due to differences between experimental flocks in factors such as breed, genetics, mature body size and nutrition during mating. Therefore, the principal is clear that when bred early, managing maternal composite ewe lambs to be as heavy as realistically feasible will improve their reproductive rate. However, in practice, the overall target liveweight at breeding for individual flocks and the cost-effectiveness of providing additional supplementary feed to certain animals within each flock pre-breeding will vary for specific management settings.

The relatively small effect of liveweight at the start of breeding on the survival of their offspring to weaning was surprising. The survival of the single- and twin-born offspring increased by only 0.4% per kg of extra liveweight at breeding over the range between 35 and 45 kg, and there were no further gains in survival of offspring if the ewe lambs achieved 45 kg or more at the start of breeding. This is the first study to directly relate the liveweight at breeding of individual ewe lambs to the survival of their offspring, but the results appear to be similar to Griffiths et al. [16] who reported that liveweight at the start of breeding had no effect on the risk of single or twin bearing ewe lambs failing to rear any offspring to lamb marking at 3 to 6 weeks of age. Mulvaney et al. [24] also reported minimal differences in offspring survival between ewe lambs that weighed 36 kg versus 42 kg at breeding. In our study, there were minimal differences in the survival of the single- and twin-born offspring for ewe lambs of the same liveweight at breeding. This suggests that nutritional management of ewe lambs during pregnancy is a more important determinant of offspring survival than liveweight at breeding, which is again consistent with Griffiths et al. [16] where liveweight change from pregnancy scanning to lambing had a much greater effect than liveweight at breeding on the risk of failing to rear any offspring. It also indicates that the differential management of the single- and twin-bearing flocks involved in our study during both pregnancy and lambing was effective at reducing the difference in offspring survival normally observed when single- and twin-bearing ewes are managed together regardless of age and breed [25,26,27,28,29,30]. Nevertheless, in the current study the survival of single- and twin-born offspring from ewe lambs was typically less than 70% and lower than 50% in triplet-born offspring. This emphasizes the importance of management during pregnancy and lambing to improve the survival rates of offspring from ewe lambs regardless of their liveweight at breeding, and more work is needed to develop management guidelines to improve offspring survival from ewe lambs.

Ewe lambs that were older at the start of breeding weaned more offspring due to both increased reproductive rate and higher survival of offspring. This is the first study to separate the effects of age and liveweight on the components of weaning rate, as they are often confounded. There was a curvilinear effect of age at breeding on reproductive rate that increased only marginally when ewe lambs were older than 8 months at the start of breeding, and there was no difference in reproductive rate between the ewe lambs that were 8.5 versus 9 months of age at the start of breeding. The ewe lambs in this dataset were not teased using vasectomized rams and the estimated date of conception was about 25 days after the start of breeding, so the absolute age at breeding beyond which there are no further gains in reproductive rate may vary between flocks. Whilst reproductive rate did not increase significantly above 8 months of age, age of breeding up to 9 months of age increased the survival of their offspring by about 4% per month regardless of their birth type. We therefore conclude that ewes that were 8 months of age at the start of breeding were sufficiently mature to become pregnant, however older ewe lambs were more capable of successfully rearing their offspring to weaning. Together with the responses to liveweight at the start of breeding, these data will contribute to whole farm economic modelling to determine the optimal liveweight and age for breeding maternal composite ewe lambs.

The age of the ewe lambs at breeding had a greater impact on offspring survival than their liveweight at breeding. The liveweight and age of ewe lambs at breeding both had significant effects on the birth weight of their offspring. It was predicted that an extra 10 kg of liveweight at breeding or one month of age at breeding independently increased the birth weight of their offspring by 0.19 and 0.14 kg, respectively. These effects of liveweight at breeding on birth weight and the quadratic relationship between birthweight and survival were similar to that observed for ewe lambs [12,30] and adult ewes regardless of breed [27,28,29]. The optimum birth weight for the offspring from ewe lambs was about 5 kg in our study, which is similar to that reported by Schreurs et al. [31] but greater than that reported by Young et al. [25] and McMillan [29]. The effects of the age of ewe lambs on birthweights have not been reported previously. Furthermore, the tendency for liveweight at breeding to influence survival was not evident when birthweight was included in the model, whereas the effect of age at breeding on survival remained even after accounting for the effects of age at breeding on birthweight. Clearly, the benefits of age at breeding on offspring survival are achieved via mechanisms additional to birthweight. These effects of age at breeding on offspring survival may be mediated via an improved mothering ability and or milk production, since it has been reported that Lacaune ewe lambs mated before 8 months of age produced significantly less milk in their first lactation than older ewe lambs [32]. This suggests that we need more detailed studies on the mechanism by which age at breeding affects offspring survival to produce a comprehensive program for managing young ewes optimally.

On average, only 3% of ewe lambs died between pregnancy diagnosis and the weaning of their offspring, which was lower than the 4.4 to 13.4% mortality reported for four cohorts of ewe lambs on three commercial farms in New Zealand [33]. The mortality of young ewes was influenced by both liveweight and age at breeding, but their effects were small. Flay et al. [33] reported no association between the pre-breeding condition score and mortality. The reason for the slightly higher mortality of ewe lambs that were heavier than 45 kg at breeding in our study is not known, but it could not be attributed to a higher proportion of twin offspring as litter size had no significant effect on dam mortality. It was also expected that mortality would be lower for ewe lambs that were older at breeding, but the linear effect of age on mortality was less than 0.25% per month. We therefore conclude that the effects of the liveweight and age at breeding of ewe lambs on their weaning rate are mainly mediated via reproductive rate and or offspring survival but not dam mortality. Furthermore, the minimal effects of liveweight at breeding on either dam mortality or mortality of their offspring suggests that breeding all ewe lambs regardless of their liveweight will not adversely impact animal welfare. The decision to select only a proportion of ewe lambs for breeding based on a minimum liveweight at breeding will depend on whether there are any adverse impacts on their reproductive performance at the subsequent breeding, which requires further investigation.

Multiple-born ewe lambs were about 3 kg lighter than single-born ewe lambs when they were bred at 6 to 9 months of age, and yet they conceived and raised more progeny even after an adjustment for the difference in liveweight or age at breeding. Some smaller studies observed no association between the ewe’s own birth type and her reproductive performance at 7 to 9 months of age [10,11,13,14]. Although the mechanisms that underpinned the effect of own birth type on reproductive performance in the current study were not tested, they are likely to be related to management and environment rather than genetics. Multiple-born and reared lambs are known to gain weight more rapidly following weaning [34], and higher growth rates from weaning to breeding have been associated with more ewes achieving estrus for a given liveweight [35]. In addition, the higher reproductive performance of these multiple-born lambs at 6 to 9 months of age in our study was not as evident at their second breeding at 19–20 months (Rosales-Nieto unpublished data). These findings may have implications for differential liveweight targets at breeding for single and multiple-born ewe lambs because comparable reproductive outcomes can be achieved at a breeding liveweight 2–3 kg less for multiple-born ewe lambs than single-born ewe lambs.

## 5. Conclusions

The present study quantified the effects of liveweight and age at breeding on the reproductive performance of more than 11,500 maternal composite ewe lambs. Ewe lambs that were heavier and older at the start of the breeding period weaned more offspring than lighter and younger ewes. The curvilinear effect of liveweight on weaning rate was driven largely by impacts on reproductive rate, whereas the age effect on weaning rate was linear and ewe lambs that were older weaned more offspring due to increases in both reproductive rate and survival of offspring. Quantifying the scale of this trade-off between liveweight and age at breeding on weaning rate and its components will assist sheep producers to make more informed decisions relating to the time of breeding ewe lambs and their nutritional management before breeding through to lambing. However, further work is needed to establish the economic optimum liveweight and age at breeding ewe lambs in different environments, including whether it is more profitable to only breed a proportion of ewe lambs based on a minimum liveweight at breeding. In the current study, the overall survival of offspring was less than 70% so more work is also needed to develop management guidelines to improve offspring survival from ewe lambs.

## Figures and Tables

**Table 1:** Number of maternal composite ewe lambs bred by maternal birth type and breeding year.

Breeding Year	Singleton	Twin	Triplet	Total
2010	79	363	76	518
2011	444	914	137	1495
2012	489	1398	338	2225
2013	367	868	151	1386
2014	352	920	117	1389
2015	311	805	79	1195
2016	345	1262	131	1738
2017	343	1135	175	1653
Total	2730	7665	1204	11,599

**Table 2:** Average liveweight and age at breeding for the whole flock and single-, twin- and triplet-born maternal composite ewe lambs from 2010 to 2017.

Breeding Year	Liveweight (kg)				Age (Days)			
	Flock	Single	Twin	Triplet	Flock	Single	Twin	Triplet
2010	43.4	47.1	42.8	42.2	222	217	221	231
2011	41.9	43.4	41.3	41.4	222	211	224	240
2012	38.0	39.2	37.8	37.2	235	224	237	244
2013	40.5	42.6	39.8	39.3	229	229	229	233
2014	41.4	44.0	40.7	39.1	226	225	226	231
2015	39.3	41.4	38.7	37.2	230	228	231	232
2016	41.0	43.7	40.5	39.0	221	212	223	230
2017	38.9	40.9	38.7	36.7	232	225	233	235
Mean	40.2	42.2 ^a^	39.7 ^b^	38.6 ^b^	228	221 ^a^	228 ^b^	235 ^c^

Different superscripts differ (*p* < 0.05) between ewe lambs born as singles, twins or triplets and comparisons only apply to the overall averages across the eight years.

**Table 3:** Average fertility rate (% ewe lambs pregnant), reproductive rate (fetuses scanned per 100 ewes bred) and weaning rate (offspring weaned per 100 ewes bred) for the whole flock and single-, twin- and triplet-born maternal composite ewe lambs from 2010 to 2017.

Breeding Year	Fertility				Reproductive Rate				Weaning Rate			
	Flock	Single	Twin	Triple	Flock	Single	Twin	Triplet	Flock	Single	Twin	Triplet
2010	58	66	57	57	95	106	93	93	58	57	59	55
2011	70	61	73	80	108	90	113	132	68	56	71	82
2012	70	64	72	72	104	92	107	106	67	58	69	73
2013	70	77	68	69	112	124	106	118	70	75	67	73
2014	74	71	74	78	116	107	118	128	83	78	85	85
2015	75	82	73	73	111	117	109	110	76	77	76	79
2016	87	88	87	85	133	132	133	131	80	66	82	91
2017	93	91	93	95	145	139	146	153	102	95	104	105
Mean	76.4	77.7	76.9	78.6	117.4	110.4 ^a^	115.8 ^b^	121.1 ^b^	76.9	69.1 ^a^	76.1 ^b^	80.1 ^b^

Different superscripts differ (*p* < 0.05) between ewe lambs born as singles, twins or triplets and comparisons only apply to the overall averages across the eight years.

**Table 4:** Average birth weight (kg) and survival to weaning (%) of all offspring and single-, twin- and triplet-born offspring from maternal composite ewe lambs from 2010 to 2017.

Breeding Year	Birth Weight				Survival			
	Flock	Single	Twin	Triplet	Flock	Single	Twin	Triplet
2010	4.42	4.96	4.31	3.04	61.3	61.3	61.7	50.0
2011	4.31	5.00	4.09	3.48	66.8	67.7	67.2	52.4
2012	4.49	5.27	4.25	3.63	68.0	67.1	71.3	48.4
2013	4.19	4.68	4.07	3.54	67.3	64.8	68.7	65.0
2014	4.64	5.13	4.53	3.87	72.0	80.7	70.5	43.7
2015	4.40	4.85	4.15	3.85	69.3	74.2	69.0	32.8
2016	3.80	4.19	3.67	3.05	62.3	61.1	64.1	44.4
2017	4.57	5.14	4.41	3.85	70.5	77.8	68.6	50.9
Mean	4.32	4.89 ^a^	4.20 ^b^	3.58 ^c^	67.7	69.5 ^a^	67.8 ^a^	48.0 ^b^

Different superscripts differ (*p* < 0.05) between offspring from ewe lambs born as singles, twins or triplets and comparisons only apply to the overall averages across the eight years.

**Table 5:** Regression coefficients (±s.e.) of Restricted Maximum Likelihood Model analysis that predicts birthweight (kg) of individual offspring from liveweight and age of maternal composite ewe lambs at breeding, birth type of ewe lamb and birth type and sex of offspring. Data represents a combined analysis across all years and all terms are significant (*p* < 0.001).

Term	Coefficient (±s.e.)
Constant ^A^	3.22 ± 0.184
Liveweight at breeding (kg)	0.0193 ± 0.00186
Age at breeding (days)	0.0045 ± 0.00061
Own birth type twin	0.09 ± 0.021
Own birth type triple	0.09 ± 0.030
Offspring birth type twin	−0.80 ± 0.019
Offspring birth type triple	−1.50 ± 0.039
Offspring female	−0.24 ± 0.015

^A^ The birth weight constant is for single-born male offspring from ewe lambs born a single.

**Table 6:** Regression coefficients (±s.e.) of General Linear Mixed Model analysis that predicts survival to weaning of individual offspring in terms of age of maternal composite ewe lambs at breeding and offspring birthweight, birth type and sex. Data represent a combined analysis across all years and data were transformed (logit). All terms are significant (*p* < 0.001), except age at breeding (*p* = 0.05).

Term	Coefficient (±s.e.)
Constant ^A^	−4.8 ± 0.51
Age at breeding (days)	0.0032 ± 0.00163
Birthweight (kg)	2.18 ± 0.173
Birthweight squared (kg)	−0.20 ± 0.020
Birth type twin	−0.31 ± 0.075
Birth type triple	−0.73 ± 0.133
Female	0.44 ± 0.054

^A^ The survival constant is for single male offspring.

## Data Availability

None of the data were deposited in an official repository, yet information can be available upon request.
